# Structural footprinting in protein structure comparison: the impact of structural fragments

**DOI:** 10.1186/1472-6807-7-53

**Published:** 2007-08-09

**Authors:** Elena Zotenko, Rezarta Islamaj Dogan, W John Wilbur, Dianne P O'Leary, Teresa M Przytycka

**Affiliations:** 1 Department of Computer Science, University of Maryland, College Park, MD 20742, USA; 2 Institute for Advanced Computer Studies, University of Maryland, College Park, MD 20742, USA; 3 National Center for Biotechnology Information, National Library of Medicine, National Institutes of Health, Bethesda, MD 20894, USA

## Abstract

**Background:**

One approach for speeding-up protein structure comparison is the *projection approach*, where a protein structure is mapped to a high-dimensional vector and structural similarity is approximated by distance between the corresponding vectors. *Structural footprinting methods *are projection methods that employ the same general technique to produce the mapping: first select a representative set of structural fragments as *models *and then map a protein structure to a vector in which each dimension corresponds to a particular model and "counts" the number of times the model appears in the structure. The main difference between any two structural footprinting methods is in the set of models they use; in fact a large number of methods can be generated by varying the type of structural fragments used and the amount of detail in their representation. How do these choices affect the ability of the method to detect various types of structural similarity?

**Results:**

To answer this question we benchmarked three structural footprinting methods that vary significantly in their selection of models against the CATH database. In the first set of experiments we compared the methods' ability to detect structural similarity characteristic of evolutionarily related structures, i.e., structures within the same CATH superfamily. In the second set of experiments we tested the methods' agreement with the boundaries imposed by classification groups at the Class, Architecture, and Fold levels of the CATH hierarchy.

**Conclusion:**

In both experiments we found that the method which uses secondary structure information has the best performance on average, but no one method performs consistently the best across all groups at a given classification level. We also found that combining the methods' outputs significantly improves the performance. Moreover, our new techniques to measure and visualize the methods' agreement with the CATH hierarchy, including the threshholded affinity graph, are useful beyond this work. In particular, they can be used to expose a similar composition of different classification groups in terms of structural fragments used by the method and thus provide an alternative demonstration of the continuous nature of the protein structure universe.

## Background

Protein structure comparison is an important tool that helps biologists understand various aspects of protein function and evolution. Unfortunately highly accurate protein structure comparison methods are computationally expensive and therefore are not suitable for large-scale analysis, such as when all pairwise comparisons have to be performed for a large number of protein structures. One approach for speeding-up protein structure comparison is the *projection approach*, where a protein structure is mapped to a vector in a high-dimensional space. Once the mapping is done, protein structure comparison is reduced to a distance computation between the corresponding vectors and therefore is very efficient. For example, it was shown [1] that once vector representations are computed it takes on average 500 seconds for a projection method to perform all pairwise comparisons among 5,024 domains. (Compare this to an estimated four months it would take DALI [2], a highly accurate protein structure comparison method, to perform the same number of pairwise comparisons.) However, the advantage of the projection approach is also one of its main limitations; namely, in the process of mapping, some structural information is lost. Furthermore, there is no agreement on what constitutes a good projection technique, and currently known projection methods [1,3-7] utilize very different approaches to the mapping construction, both in terms of which structural information is included and how this information is integrated to produce a vector representation.

Recently, Zotenko *et al*. [1] performed a comprehensive comparison of projection methods in the context of two typical applications for such methods, high-throughput protein structure comparison and classification. The authors found that the SSEF method [1] performed the best in their tests, followed closely by the LFF method [5]. Both methods use the same general approach, which we call *structural footprinting*, to construct the mapping: (i) select a representative set of structural fragments as *models*, (ii) map a structure to a *structural footprint*, a vector in which each dimension corresponds to a particular model and "counts" the number of times the model appears in the structure. (Since structural fragments are not discrete objects, a count of one is distributed among one or several most similar models weighted by the precision with which the model is reproduced in the structure.) While both methods use the same strategy to integrate the structural information, they differ substantially in the type of structural fragments used as models and their representation. The LFF method [5] uses a pair of backbone segments (each ten residues long) as a structural fragment whose conformation is described by a set of 100 inter-atomic distances between the corresponding *C_α _*atoms. The SSEF method [1] uses a triplet of secondary structure elements (throughout the paper we use SSE to refer to a secondary structure element) as a structural fragment whose conformation is captured by a set of pairwise angles and distances between the corresponding SSE vectors. Even though the comparison results of Zotenko *et al*. [1] show that the structural footprinting is an adequate approach to the mapping construction, we are not aware of any systematic study that evaluates the effect of the choice of structural fragments on the ability of a structural footprinting method to detect different types of structural similarity.

The main objective of this work is to explore in detail the dependence of a structural footprinting method on the set of structural fragments it selects to model the structure and their representation. Towards this end we focus our attention on three structural footprinting methods that vary significantly in their selection of models. To complement the LFF and SSEF methods described above, we have designed a structural footprinting method that uses contiguous segments (thirty-two residues long) of protein backbone as structural fragments; we call this method the SEGF method. The conformation of a backbone segment is captured by a set of fourteen shape descriptors introduced by Rogen *et al*. [3,8]. These descriptors build upon a geometric invariant inspired by the *writhing number *of a closed space curve [9], a concept from Knot Theory. As opposed to the common geometric invariants such as angles and distances, the fourteen shape descriptors lack intuitive interpretation, with each descriptor being a function of many factors (see Methods).

We benchmarked the methods' performance against the CATH database [10]. CATH is an hierarchical classification of protein structures, where protein domains are classified into groups at the Class, Architecture, Topology (Fold), and Homologous Superfamily levels. Members of the same homologous superfamily group share a clear common evolutionary origin supported either by significant sequence similarity or significant structural and functional similarity, and several superfamilies are grouped into topology (fold) groups based on significant structural similarity. The architecture level further groups proteins based on coarse topological organization of secondary structure elements. Finally, the class level groups proteins according to secondary structure element content: mainly *α*, mainly *β*, mixed *α *and *β*, or small structures.

In the first set of experiments we compared the methods' ability to detect structural similarity characteristic of evolutionarily related structures, i.e., structures within the same CATH superfamily. In a recent study [11], Reeves and colleagues analyzed the extent and nature of structural diversity across different superfamilies in the CATH database. In particular, it was shown that some superfamilies, especially those from layered architectures such as mainly *β *or *α*-*β *sandwiches, are much more structurally diverse than others. Moreover, the repertoire of structural changes is very rich, ranging from changes in conformation of the loop regions, to changes in the orientation of secondary structure elements, to insertion/deletion of secondary structures or even whole super-secondary structure motifs. Thus, studying the relative performance of the methods across different superfamilies allowed us to observe the relative strengths and weaknesses of the methods in a variety of settings and to propose two strategies to combine the methods to achieve a better performance. We showed that combination methods provide a significant improvement in performance. Even though the method that uses the SSE information has the best performance on average, combining the methods allows better handling of the whole spectrum of structural variability exhibited by various CATH classification groups.

Recently several groups [12-14] demonstrated the existence of meaningful structural relationships between protein domains classified in different folds. Harrison and colleagues [12], for example, introduced a measure of *gregariousness*, where the gregariousness of a fold quantifies how many other folds have a significant structural overlap with it. The presence of common structural motifs, often on a level of super-secondary structure elements, emerged as one of the main reasons for these inter-fold similarities. As a structural footprinting method measures structural similarity based on the presence/absence of common structural fragments, inter-fold similarities of this kind should be prominent in the method's view of the protein structure universe. Therefore, in the second set of experiments we tested how the method's definition of structural similarity extends beyond the Superfamily level and whether it agrees with the boundaries imposed by the classification groups at the Class, Architecture, and Fold levels of the hierarchy. To study these similarities in a systematic way we defined an *affinity score *of one superfamily towards the other, which measures how well the method retrieves members of the second superfamily using the members of the first superfamily as queries. We developed a set of techniques that allowed us to summarize the affinity scores for a particular method to expose the agreement between the method and the CATH classification hierarchy.

## Results and discussion

### The evaluation procedure

In this work we used the CATH database (version 2.6 released on April 2005) for benchmarking purposes. We used a set of non-redundant domains (a total of 5,588 domains) as our set of database domains. From these we selected a set of 133 well-populated superfamilies that span 55 folds, 17 architectures and 3 classes (see Methods). Given a structural footprinting method and a well-populated superfamily, every member of the superfamily was used by the method as a query to rank the remaining database domains. We then used the *ROC*_300 _scores [15], which measure to what extent the positives (the remaining members of the superfamily) precede the negative results (domains in folds different than that of the query), to quantify the method's ability to retrieve the other members of a superfamily given one member as a query.

In what follows we use the CATH numbering system to refer to individual superfamilies, folds, architectures, and classes. The CATH number of a classification group encodes its position within the hierarchy. Thus, for example, the 3.30.70.100 superfamily is in the 3.30.70 fold group, in the 3.30 architecture group, and in the 3 class group.

### Structural similarity at the CATH Superfamily level

**Figure 1:**
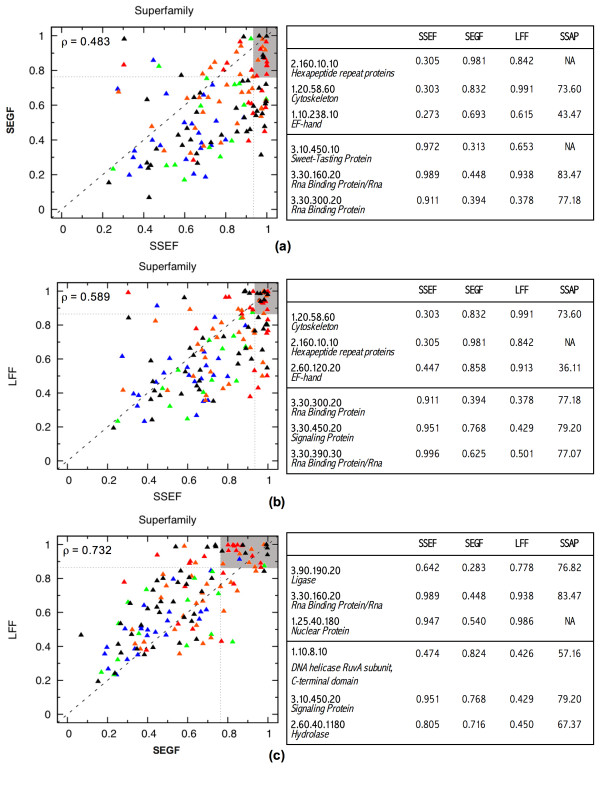
**The relative performance of the methods, comparing the *ROC*_300 _score across superfamilies**. There is one scatter plot per pair of methods: SSEF and SEGF **(a)**, SSEF and LFF **(b)**, and SEGF and LFF **(c)**. Each superfamily is a point on the plot with the coordinates being the *ROC*_300 _scores of the corresponding methods. For a pair of methods, groups whose position significantly deviates from the main diagonal are examples of relative strength and weakness of the methods; for every pair of methods, six superfamilies that deviate the most from the diagonal are listed in the table adjacent to the plot. The superfamilies are colored according to the minimum SSAP score for a pair of domains in the superfamily as reported by the DHS database [23]: blue for scores in (0.0, 53.44], green for scores in (53.44, 63.32], orange for scores in (63.32, 73.48], and red for scores in (73.48, 100.00]. The SSAP (Sequential Structure Alignment Program) method [24] is a robust protein structure alignment method that uses a double dynamic programming strategy to align protein structures. The SSAP score measures the structural similarity on a scale from 100.0 (the most similar) to 0.0 (the least similar). Our chosen threshold values, 53.44, 63.32, and 73.48, correspond to the 25th, 50th, and 75th percentile respectively. The superfamilies for which the SSAP scores are not available are colored black. The correlation between the performance of every pair of methods is captured by Pearson correlation coefficient which is shown in upper left corner of the corresponding plots.

Even though the SSEF method has the best performance on average (see Table 1 and Additional file 1), no method performs consistently the best over all superfamilies. Figure 1 shows the *ROC*_300 _scores as a scatter plot; there is one plot per pair of methods; each superfamily is a point on the plot with the coordinates being the *ROC*_300 _scores of the corresponding methods. The performance of the methods is poorly correlated, especially that of the SSEF and SEGF methods. The poor correlation can be attributed to the fact that the methods capture different aspects of protein structure in their footprints. Thus structural differences between the members of a superfamily may "confuse" some methods more than others and the amount of confusion depends on how these structural differences affect the structural footprint produced by the method.

**Table 1:** Average *ROC*_300 _scores for combined methods. The average *ROC*_300 _scores obtained over a range of combination strategies: the original methods, voting with all three methods, and linear combination of similarity scores. (Individual *ROC*_300 _scores are given in the supplementary material [see Additional file 1].)

SSEF	SEGF	LFF	voting	linear combination			
				SSEF+SEGF+LFF	SSEF+SEGF	SEGF+LFF	SEGF+LFF
0.750	0.581	0.665	0.774	0.814	0.798	0.789	0.677

To illustrate this point let us consider three outliers in Figure 1, superfamilies for which the performance of one method is quite different from that of another, the 1.20.58.60 (*Cytoskeleton*), 3.30.300.20 (*Rna Binding Protein*), and 3.30.450.20 (*Signaling Protein*) superfamilies.

**Figure 2:**
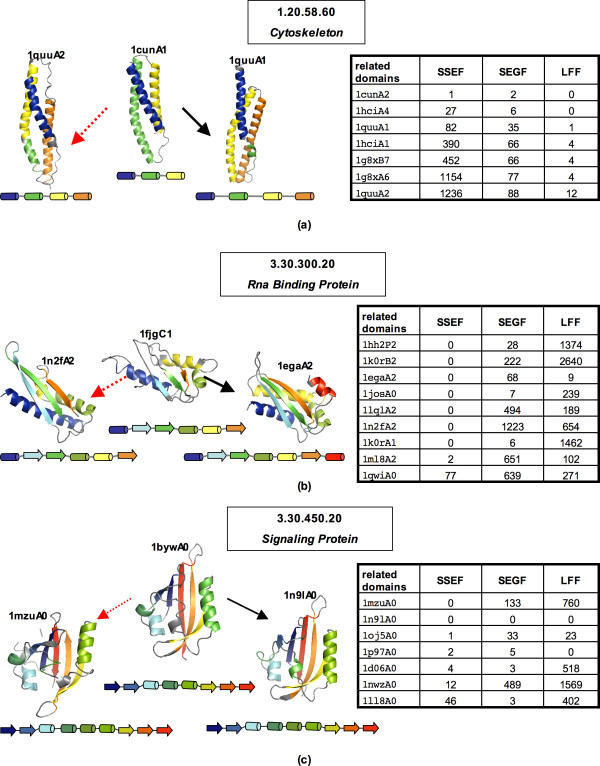
**Outliers, the 1.20.58.60, 3.30.300.20, and 3.30.450.20 superfamilies**. **(a) **The 1.20.58.60 (*Cytoskeleton*) superfamily; In the table to the right, for each database domain related to the query 1cunA1, we show the number of errors encountered before the domain is retrieved. Both the SEGF and LFF methods retrieve all seven related domains before the 300th error. (In this case, any domain in a fold group other than 1.20.58 is counted as an error.) In contrast, the SSEF method retrieves only 1cunA2, 1hciA4, and 1quuA1. The structure of the query domain 1cunA1 and two related domains are shown on the left, colored according to secondary structure assignments and also schematically represented by diagrams adjacent to the structures. The secondary structure assignment was computed using the DSSP (Dictionary of Protein Secondary Structure) program [25]. **(b) **The 3.30.300.20 (*Rna Binding Protein*) superfamily; Given the 1fjgC1 Domain as a query, the SSEF method retrieves all nine related domains before the 300th error. On the other hand, the SEGF and LFF method retrieve only five related domains. The ranking of the related domains is summarized in the table to the right. The structure of the query domain 1fjgC1 and two related domains are shown on the left, colored according to secondary structure assignments and also schematically represented by diagrams adjacent to the structures. **(c) **The 3.30.450.20 (*Signaling Protein*) superfamily, an example where the LFF method performs worse than the other two methods; Given the 1bywA0 domain as a query, the SSEF method retrieves all seven related domains, while the SEGF method retrieves six related domains before the 300th error. On the other hand, the LFF method retrieves only three related domains. The ranking of the related domains is summarized in the table to the right. The structure of the query domain 1bywA0 and two related domains are shown on the left, colored according to secondary structure assignments and also schematically represented by diagrams adjacent to the structures. The protein structures were rendered using PyMOL [26].

The poor performance of the SSEF method on the 1.20.58.60 superfamily can be partially attributed to variability in secondary structure assignment as shown in Figure 2(a). Since the second helix in the 1quuA2 domain is split into two helices, the 1quuA2 domain has four SSE triplets that participate in the footprint construction, while the 1cunA1 domain has only one such triplet. In this case, the structural change that produced an additional SSE is small and therefore both the SEGF and LFF methods perform well since they do not use secondary structure information. In general, the SSEF method is most sensitive to structural changes that affect the number and/or relative orientation of SSEs.

In contrast, the structural variability exhibited by the members of the 3.30.300.20 superfamily does not affect the performance of the SSEF method, but it does affect the other two methods. As shown in Figure 2(b), the members of this superfamily have approximately the same number of SSEs, and they are oriented in roughly the same way.

The 3.30.450.20 (*Signaling Protein*) superfamily contains structural representatives of the PAS domain, a family of sensor protein domains involved in signal transduction [16]. The common fold shared by the PAS domains is flexible to accommodate binding of a large variety of co-factors, which allows PAS domains to serve as input modules in proteins that sense light, redox potential, and other stimuli [17]. As shown in Figure 2(c), in this case the structural variability characteristic of the members of this superfamily confuses the LFF method more than the other two methods.

It is reasonable to assume that for structurally conserved superfamilies all three methods would perform well. To check this hypothesis we color coded the points in the scatter plots of Figure 1 according to the structural diversity of the corresponding superfamilies, where red color denotes the most structurally conserved superfamilies and blue the least structurally conserved superfamilies. Even though the concentration of the red points in the upper-right corner is clearly visible on all three plots, there are structurally conserved superfamilies for which one or more methods do not perform well. This can happen when a small structural change triggers a big change in the structural footprint produced by the method; consider for example performance of the SSEF method on the 1.20.58.60 superfamily discussed above. Another reason for a poor performance of a method on a structurally conserved superfamily is its inability to distinguish between the members of the superfamily and members of other superfamilies that are composed of similar structural fragments but have different overall structure.

### Combining the methods

Can we take advantage of variation in performance of the methods across different superfamilies, i.e. can the output of the methods be combined in such a way as to leverage their relative strengths? To answer this question we have studied two combination strategies: voting and linear combination of similarity scores. Given a query domain, both strategies use the original similarity scores to produce a new ranking of database domains. In voting, each method's similarity scores are first used to rank the database domains. The new score of a database domain is determined by averaging the domain's positions in the three original rankings, with ties being resolved arbitrarily. In linear combination, a new structural similarity score between the query and a database domain is defined as a linear combination of the original similarity scores, where the optimal coefficients are learned with a Support Vector Machine (SVM) [18] from a set of positive and negative examples (see Methods). The new similarity score is then used to rank the database domains.

As shown in Table 1, the average *ROC*_300 _scores increase from 0.750 (the SSEF method), to 0.774 (the voting combination strategy), to 0.814 (the linear combination strategy). Even with the simple voting strategy we obtain an improvement of 0.024 over the best (on average) method; the introduction of weights (in linear combination strategy) further improves the performance by 0.040. We used the *binomial sign test for two dependent samples *[19] to evaluate the statistical significance of improvements due to combination. This test can be applied to evaluate whether a number of superfamilies on which one method outperforms the other differs significantly from what would be expected by chance. We found that both combination strategies significantly improve over the SSEF method: the improvement due to voting has a p-value of 3.35*e*-02 and improvement due to linear combination has a p-value of 1.43*e*-15.

The success of a combination strategy largely depends on how consistent are the methods in their ranking of false positives. The combination is most effective when the methods disagree on their ranking of false positive domains, i.e., false positive domains ranked near the top by one method are ranked near the bottom by other methods. Thus the success of a combination strategy is a function of the methods being combined. To find out which pair of methods are the most complementary, i.e., their combination gives the best results, we repeated the linear combination experiments for all pairs of methods. The outcomes of these experiments (see Table 1 under SSEF+SEGF, SSEF+LFF, and SEGF+LFF) indicate that combination of the SSEF and SEGF methods gives the best results. This outcome demonstrates that the stand-alone performance is of lesser importance for combination purposes. Indeed, while the SEGF method is the weakest among the three methods, its performance is the least correlated with that of the SSEF method (see the performance correlation values in Figure 1).

The success of the combination strategies supports our hypothesis that the methods are indeed complementary i.e., no single approach is able to deal with a full spectrum of structural variability exhibited by different superfamilies. Moreover, we observe that the combination of two least correlated methods (SSEF+SEGF) is better than SSEF+LFF or LFF+SEGF.

### Structural similarity at the CATH Class, Architecture, and Fold levels

So far we have evaluated whether the methods' definition of structural similarity agrees with the CATH hierarchy at the Superfamily level. Here we extend this evaluation to the higher (Class, Architecture, and Fold) levels of the hierarchy by studying the similarities, as seen by a particular method, among different superfamilies. To quantify these similarities we introduce an *affinity score *between a pair of superfamilies which measures how well the method retrieves the members of the second superfamily using members of the first superfamily as queries. More formally, the affinity score of superfamily *A *towards superfamily *B *is an average ROC score over all rankings of database domains produced by the method with the members of A as queries, where the positives are the remaining members of A plus members of B and the negatives are all other domains in the database. It should be noted that affinity scores are not symmetric, i.e., affinity of *A *towards *B *is not necessarily the same as affinity of *B *towards *A*.

**Figure 3:**
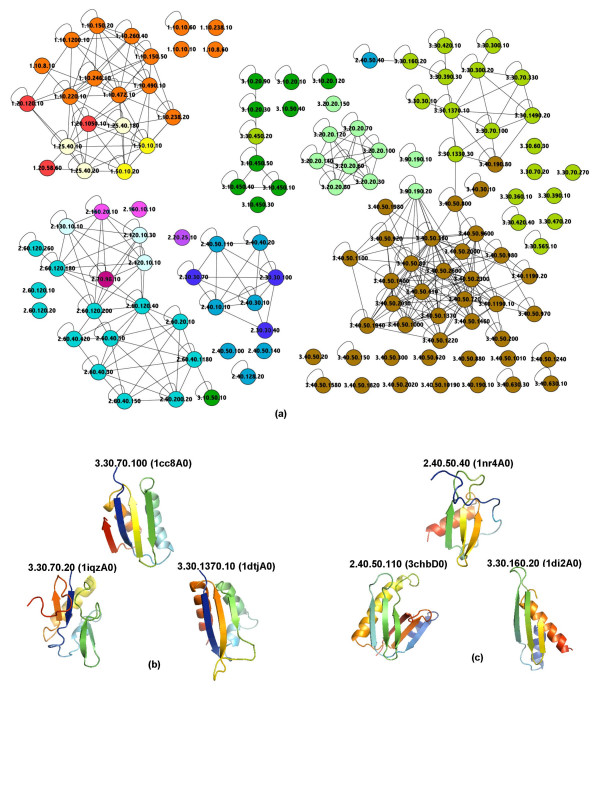
**The thresholded affinity graph for the SSEF method**. **(a) **The thresholded affinity graph for the SSEF method, where vertices are superfamilies in our dataset of 133 well populated CATH superfamilies and there is an edge between a pair of superfamilies if and only if both affinity scores are above a certain threshold. The threshold is such that 75% of self affinity scores, the affinity score of a superfamily to itself, are above this value. The superfamilies are color-coded according to the architecture group to which they belong. (Affinity graphs for the SEGF and LFF methods are given in the supplementary material [see Additional file 2].) **(b)–(c) **Structures of representative domains for some superfamilies involved in interesting interconnection patterns in the affinity graph. The affinity graph was drawn using Cytoscape [27]. The protein structures were rendered using PyMOL [26].

Affinity scores can be used to construct an *affinity graph *A
 MathType@MTEF@5@5@+=feaafiart1ev1aaatCvAUfKttLearuWrP9MDH5MBPbIqV92AaeXatLxBI9gBamrtHrhAL1wy0L2yHvtyaeHbnfgDOvwBHrxAJfwnaebbnrfifHhDYfgasaacH8akY=wiFfYdH8Gipec8Eeeu0xXdbba9frFj0=OqFfea0dXdd9vqai=hGuQ8kuc9pgc9s8qqaq=dirpe0xb9q8qiLsFr0=vr0=vr0dc8meaabaqaciaacaGaaeqabaWaaeGaeaaakeaaimaacqWFaeFqaaa@3821@ for a set of superfamilies under investigation; the affinity graph is a complete directed graph with weighted edges, where vertices are superfamilies and weights are affinity scores. The affinity structure of this set of superfamilies can be exposed by a simplified and thresholded representation of the affinity graph, A
 MathType@MTEF@5@5@+=feaafiart1ev1aaatCvAUfKttLearuWrP9MDH5MBPbIqV92AaeXatLxBI9gBamrtHrhAL1wy0L2yHvtyaeHbnfgDOvwBHrxAJfwnaebbnrfifHhDYfgasaacH8akY=wiFfYdH8Gipec8Eeeu0xXdbba9frFj0=OqFfea0dXdd9vqai=hGuQ8kuc9pgc9s8qqaq=dirpe0xb9q8qiLsFr0=vr0=vr0dc8meaabaqaciaacaGaaeqabaWaaeGaeaaakeaaimaacqWFaeFqaaa@3821@(*τ*), where there is an undirected edge between a pair of superfamilies if and only if both affinity scores are above the threshold *τ*. Figure 3(a) shows such a graph for the SSEF method [see Additional file 2 for affinity graphs for the other methods]. By relating the interconnection patterns in this graph with the known CATH classification we can quickly detect where the method's definition of structural similarity violates the boundaries imposed by the hierarchy; consider, for example, an interconnection pattern where a superfamily is connected to a superfamily outside its fold while having very few connections to superfamilies within its fold. For the SSEF method (see Figure 3(a)), one such violation is around the 3.30.70.100 superfamily. The 3.30.70.100 superfamily is connected to the 3.30.1370.10 superfamily but not to the 3.30.70.20 or 3.30.70.270 superfamilies within the same fold. (The structures of representative domains are given in Figure 3(b).) A similar example is around the 2.40.50.40 superfamily; indeed, this superfamily's only connection is to 3.30.160.20. In this case the SSEF method is unable to distinguish between two different orientations of the last *α*-helix relative to the *β*-sheet in 2.40.50.40 and 3.30.160.20 superfamilies and therefore deems the 2.40.50.40 superfamily to be more similar to the 3.30.160.20 than to say the 2.40.50.110 superfamily. (See Figure 3(c) for structures of representative domains.)

We can also use affinity scores to quantify the agreement between the method and the hierarchy for individual classification groups. Given a classification group, we say that there is a perfect agreement between the method and the hierarchy if, for every member superfamily, within-the-group affinity scores (affinity scores of the superfamily towards other members of the group) are higher than outside-the-group affinity scores (affinity scores of the superfamily towards superfamilies in other groups). The agreement is rarely perfect; thus we measure the amount of agreement (see Methods) for every superfamily within the group and set the degree of agreement for the group to be an average of these values. The agreement values range from 0.0 to 1.0, where 0.0 corresponds to the lowest agreement. In general, an agreement value close to one means that from the method's perspective the corresponding classification groups are structurally isolated from other groups, i.e., the composition of its member protein domains in terms of structural fragments used by the method to model the structure is quite different from that of domains in other groups.

**Figure 4:**
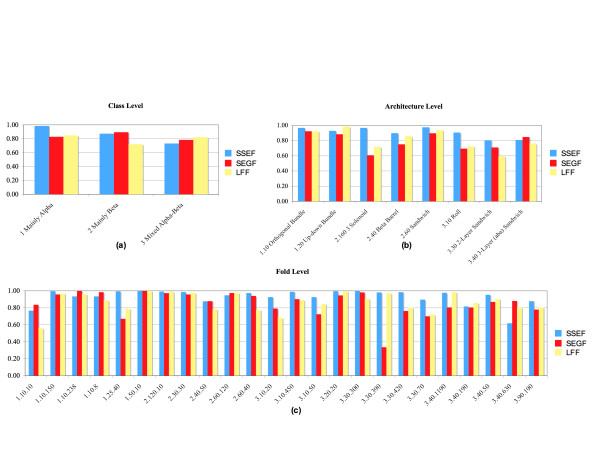
**The agreement between the methods and the CATH hierarchy**. Bar plots showing the degree of agreement between the methods' definition of structural similarity and the CATH hierarchy.

Figure 4 shows the agreement values for groups that are present in our set of well-populated superfamilies. For a given structural footprinting method, the variation of agreement values across the groups at the same classification level is clearly visible. For the SSEF method, for example, the 3.30 (*2-Layer Sandwich*) and 3.40 (*3-Layer (aba) Sandwich*) architectures have the lowest agreement values (0.806 and 0.808 respectively), while the 1.10 (*Orthogonal Bundle*), 2.160 (*3 Solenoid*), and 2.60 (*Sandwich*) architectures have the highest agreement values (0.968, 0.969, and 0.976 respectively). We observe that the variation in agreement values measured for the SSEF method is consistent to some extent with the findings of Harrison and colleagues [12]. The authors found that folds in some densely populated architectures, such as *α*-*β *Sandwiches, are particularly gregarious, while folds in some sparsely populated architectures, such as *α *Solenoid, *β *3 Solenoid, and *β *Propellers, are quite structurally isolated.

Once again we observe that no one method has the highest agreement with the CATH hierarchy over all groups at a given classification level. But on average (see Table 2) the SSEF method agrees the most with the CATH hierarchy at all levels. This not surprising given that the Class, Architecture, and Fold levels of the CATH hierarchy are defined in terms of secondary structure. Therefore, a method that captures the relationship between secondary structure elements in its footprint is expected to perform better at detecting relationship at these levels. We also observe that all three methods achieve the highest average agreement values at the Fold level, which is not surprising since the Fold level groups structurally similar superfamilies.

**Table 2:** Average agreement with CATH. Average agreement with CATH classification groups at a given classification level for the three methods.

	SSEF	SEGF	LFF
Class	0.864	0.836	0.798
Architecture	0.908	0.813	0.790
Fold	0.931	0.853	0.869

## Conclusion

In this work we evaluated the effect of the model selection process on the ability of structural footprinting methods to detect various types of structural similarity. Towards this end we evaluated three methods – the SSEF, SEGF, and LFF methods – that vary greatly in terms of what structural fragments are chosen to model the structure and their representation. In our first set of experiments we studied the effect of the structural diversity exhibited by members of well-populated superfamilies in the CATH database on the methods' ability to retrieve other members of the group given one member as a query. We found that there is a large variation in performance both across the superfamilies and across the methods. This is consistent with the findings of Reeves and colleagues [11] that some superfamilies are more structurally diverse than others. Poor correlation in performance among different methods supported the hypothesis that methods are indeed complementary in the following sense: there are types of structural variation that impact some methods to a greater extent than others. We were able to demonstrate the interplay between the nature of the structural variation and the performance of individual methods by looking in depth at several outliers, superfamilies with the most pronounced difference in performance across the methods.

To exploit the complementarity of the methods we tested two strategies, voting and linear combination of similarity scores, to combine the methods' output. We found that both strategies result in significant improvement in average performance over the best method, the SSEF method, with the linear combination strategy yielding the biggest improvement. Thus, by using a linear combination of the three similarity scores to rank database proteins we were able to improve the average *ROC*_300 _scores from 0.750 (the best average score achieved by a stand-alone method) to 0.814. We next tested which pair of methods is best suited for combination and found that combining the SSEF and SEGF methods gives the best results. This is interesting since the LFF method has a significantly better performance on average than the SEGF method and therefore one might expect that the pair of SSEF and LFF would be the winner. Thus, we conclude that the ability to reverse each others' bad decisions is more important than stand-alone performance for combination purposes.

Next we studied whether the methods' definition of structural similarity agrees with the higher (Class, Architecture, and Fold) levels of the CATH hierarchy. Towards this end, we introduced an affinity score as a measure of structural similarity, as seen by a particular method, between a pair of superfamilies. We visualized the affinity scores among well-populated superfamilies by a thresholded affinity graph, where there is a vertex for each superfamily and there is an edge between a pair of superfamilies if both affinity scores are above a certain threshold. By comparing the position of a particular superfamily in the graph to its known CATH classification we were able to recover several interesting structural similarities between superfamilies in diffierent Fold and even Class levels. We also used the affinity scores to quantify the agreement between a particular method and the CATH hierarchy for individual classification groups. The agreement values allowed us to identify classification groups that are structurally isolated from other groups and to compare the agreement with the CATH hierarchy across diffierent methods. Once again we observed that no one method has the highest agreement values across all groups at a given classification level but on average the SSEF method agrees the most with the hierarchy.

Since a structural footprinting method measures structural similarity based on presence/absence of common structural fragments, we believe that affinity scores produced by the method and their analysis techniques employed in our work can be useful beyond understanding the specifics of the method. In particular, the techniques can easily expose a similar composition of diffierent groups in terms of structural fragments used by the method and thus provide an alternative view of the continuum of the protein structure universe.

## Methods

### Data sets

We used the CATH classification database version 2.6 (released on April, 2005). To create a set of database domains, we downloaded a list of non-redundant domains filtered at 35% sequence identity from the CATH classification database web-site. We excluded from the list domains for which a valid footprint could not be produced by one or more methods, which resulted in a dataset with 5,588 domains. As the SSEF method uses triplets of SSEs and the SEGF method uses backbone segments thirty-two residues long, we excluded from the original dataset of 6,003 domains 383 domains with fewer than three SSEs or shorter than thirty-two residues. We further removed 32 domains that do not contain a single valid structural fragment for either SSEF or for SEGF.

The set of database domains contains members from 1,416 superfamilies. From these we selected a set of well-populated superfamilies, superfamilies that satisfy the following constraints: (i) the superfamily has at least five members in the set of database domains and (ii) the superfamily is not the only superfamily in its fold. There are 133 superfamilies that satisfy the above constraints. These superfamilies contain 2,348 domains and span 55 folds, 17 architectures, and 3 classes.

### The SEGF Method

#### Structural fragments and their representation

We use a contiguous segment (thirty-two residues long) of protein backbone as a structural footprint. The protein backbone is viewed as a polygonal line passing through the C-*α *atoms whose conformation is captured by a set of fourteen shape descriptors, a subset of the thirty shape descriptors originally used by Rogen et al. [3,8]. The shape descriptors are various combinations of an *average crossing number*, a geometric invariant that captures the relative orientation of two oriented line segments. In what follows we first describe the average crossing number invariant and then show how the fourteen shape descriptors are constructed using this invariant as a building block.

**Figure 5:**
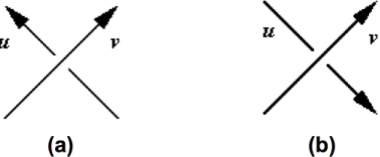
**Determining the value of an overcrossing**. When projection of a pair of oriented line segments results in an overcrossing, its value is determined by the right-hand rule involving the projection direction and directions of projected line segments. Here the projection direction is from the page to the reader. **(a) **The value of this overcrossing is +1 because the bottom line segment (*u*) is in the counterclockwise direction from the upper line segment (*v*). **(b) **The value of this overcrossing is -1 because the bottom line segment (*u*) is in the clockwise direction from the upper line segment (*v*).

**Figure 6:**
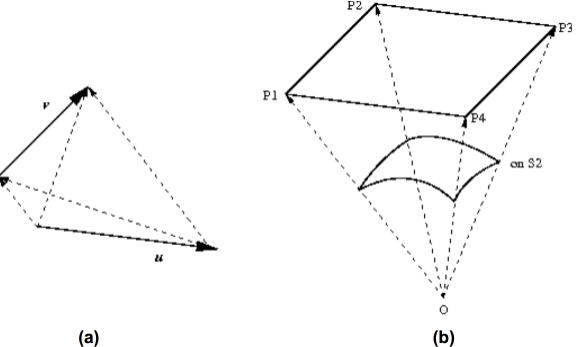
**Computing the average crossing number**. **(a) **Projection directions that result in one overcrossing are parallel to vectors of the form *t*_*v *_- *t*_*u*_, where *t*_*u *_is on *u *and *t*_*v *_on *v*. **(b) **Those directions trace a parallelogram *P *= *P*_1_*P*_2_*P*_3_*P*_4_, where *P*_1 _= *v*^*sp *^- *u*^*sp*^, *P*_2 _= *v*^*ep *^- *u*^*sp*^, *P*_3 _= *v*^*sp *^- *u*^*ep *^and *P*_4 _= *v*^*ep *^- *u*^*ep*^. The average crossing number equals the signed area of *P *projected on *S*_2 _and normalized by half of the area of *S*_2_, which can be computed using tools of Spherical Geometry [28].

Given an oriented line segment *u*, we will denote by *u*^*sp *^the coordinates of *u*'s start point and by *u*^*ep *^the coordinates of *u*'s end point. When a pair of segments, *u *and *v*, is projected on a plane it produces either zero or one overcrossing. When one overcrossing is produced, it is assigned a value of +1 or -1 as shown in Figure 5. Thus with every projection direction we can associate a value of either +1, -1, or 0 (no overcrossing). The average crossing number between two oriented line segments is the above value averaged over all possible projection directions, projection directions being points on the unit sphere *S*_2_. The value of an overcrossing, +1 or -1, is the same for all projection directions that result in an overcrossing. Moreover, projection directions that result in one overcrossing are exactly those that are parallel to vectors of the form *t*_*v *_- *t*_*u*_, where *t*_*u *_is a point on *u *and *t*_*v *_is a point on *v*. The above two facts allow us to express the average crossing number as signed area of a certain parallelogram projected on *S*_2 _and normalized by half of the area of *S*_2 _(half since there is an equivalent parallelogram of directions that correspond to vectors parallel to vectors of the form *t*_*u *_- *t*_*v*_) as shown in Figure 6. The sign of the average crossing number is equal to the sign of (*v*^*sp *^- *u*^*sp*^)^*T*^(*v × u*). Note that the range of this invariant is the closed interval [-1, 1].

Let us denote by *Wr*(*u*, *v*) the average crossing number between two oriented line segments *u *and *v*. Given a polygonal line consisting of *r *(in our case *r *= 31) oriented line segments {*u*_1_,...,*u*_*r*_} the fourteen shape descriptors are constructed in the following way:

I(1,2)=∑0≤i1<i2≤rWr(ui1,ui2)
 MathType@MTEF@5@5@+=feaafiart1ev1aaatCvAUfKttLearuWrP9MDH5MBPbIqV92AaeXatLxBI9gBaebbnrfifHhDYfgasaacH8akY=wiFfYdH8Gipec8Eeeu0xXdbba9frFj0=OqFfea0dXdd9vqai=hGuQ8kuc9pgc9s8qqaq=dirpe0xb9q8qiLsFr0=vr0=vr0dc8meaabaqaciaacaGaaeqabaqabeGadaaakeaacqWGjbqsdaWgaaWcbaGaeiikaGIaeGymaeJaeiilaWIaeGOmaiJaeiykaKcabeaakiabg2da9maaqafabaGaem4vaCLaemOCaiNaeiikaGIaemyDau3aaSbaaSqaaiabdMgaPjabigdaXaqabaGccqGGSaalcqWG1bqDdaWgaaWcbaGaemyAaKMaeGOmaidabeaakiabcMcaPaWcbaGaeGimaaJaeyizImQaemyAaKMaeGymaeJaeyipaWJaemyAaKMaeGOmaiJaeyizImQaemOCaihabeqdcqGHris5aaaa@4E1B@

I|1,2|=∑0≤i1<i2≤r|Wr(ui1,ui2)|
 MathType@MTEF@5@5@+=feaafiart1ev1aaatCvAUfKttLearuWrP9MDH5MBPbIqV92AaeXatLxBI9gBaebbnrfifHhDYfgasaacH8akY=wiFfYdH8Gipec8Eeeu0xXdbba9frFj0=OqFfea0dXdd9vqai=hGuQ8kuc9pgc9s8qqaq=dirpe0xb9q8qiLsFr0=vr0=vr0dc8meaabaqaciaacaGaaeqabaqabeGadaaakeaacqWGjbqsdaWgaaWcbaGaeiiFaWNaeGymaeJaeiilaWIaeGOmaiJaeiiFaWhabeaakiabg2da9maaqafabaGaeiiFaWNaem4vaCLaemOCaiNaeiikaGIaemyDau3aaSbaaSqaaiabdMgaPjabigdaXaqabaGccqGGSaalcqWG1bqDdaWgaaWcbaGaemyAaKMaeGOmaidabeaakiabcMcaPiabcYha8bWcbaGaeGimaaJaeyizImQaemyAaKMaeGymaeJaeyipaWJaemyAaKMaeGOmaiJaeyizImQaemOCaihabeqdcqGHris5aaaa@5269@

I(1,2)(3,4)=∑0≤i1<i2<i3<i4≤rWr(ui1,ui2)Wr(ui3,ui4)
 MathType@MTEF@5@5@+=feaafiart1ev1aaatCvAUfKttLearuWrP9MDH5MBPbIqV92AaeXatLxBI9gBaebbnrfifHhDYfgasaacH8akY=wiFfYdH8Gipec8Eeeu0xXdbba9frFj0=OqFfea0dXdd9vqai=hGuQ8kuc9pgc9s8qqaq=dirpe0xb9q8qiLsFr0=vr0=vr0dc8meaabaqaciaacaGaaeqabaqabeGadaaakeaacqWGjbqsdaWgaaWcbaGaeiikaGIaeGymaeJaeiilaWIaeGOmaiJaeiykaKIaeiikaGIaeG4mamJaeiilaWIaeGinaqJaeiykaKcabeaakiabg2da9maaqafabaGaem4vaCLaemOCaiNaeiikaGIaemyDau3aaSbaaSqaaiabdMgaPjabigdaXaqabaGccqGGSaalcqWG1bqDdaWgaaWcbaGaemyAaKMaeGOmaidabeaakiabcMcaPiabdEfaxjabdkhaYjabcIcaOiabdwha1naaBaaaleaacqWGPbqAcqaIZaWmaeqaaOGaeiilaWIaemyDau3aaSbaaSqaaiabdMgaPjabisda0aqabaGccqGGPaqkaSqaaiabicdaWiabgsMiJkabdMgaPjabigdaXiabgYda8iabdMgaPjabikdaYiabgYda8iabdMgaPjabiodaZiabgYda8iabdMgaPjabisda0iabgsMiJkabdkhaYbqab0GaeyyeIuoaaaa@6667@

I|1,2|(3,4)=∑0≤i1<i2<i3<i4≤r|Wr(ui1,ui2)|Wr(ui3,ui4)
 MathType@MTEF@5@5@+=feaafiart1ev1aaatCvAUfKttLearuWrP9MDH5MBPbIqV92AaeXatLxBI9gBaebbnrfifHhDYfgasaacH8akY=wiFfYdH8Gipec8Eeeu0xXdbba9frFj0=OqFfea0dXdd9vqai=hGuQ8kuc9pgc9s8qqaq=dirpe0xb9q8qiLsFr0=vr0=vr0dc8meaabaqaciaacaGaaeqabaqabeGadaaakeaacqWGjbqsdaWgaaWcbaGaeiiFaWNaeGymaeJaeiilaWIaeGOmaiJaeiiFaWNaeiikaGIaeG4mamJaeiilaWIaeGinaqJaeiykaKcabeaakiabg2da9maaqafabaGaeiiFaWNaem4vaCLaemOCaiNaeiikaGIaemyDau3aaSbaaSqaaiabdMgaPjabigdaXaqabaGccqGGSaalcqWG1bqDdaWgaaWcbaGaemyAaKMaeGOmaidabeaakiabcMcaPiabcYha8jabdEfaxjabdkhaYjabcIcaOiabdwha1naaBaaaleaacqWGPbqAcqaIZaWmaeqaaOGaeiilaWIaemyDau3aaSbaaSqaaiabdMgaPjabisda0aqabaGccqGGPaqkaSqaaiabicdaWiabgsMiJkabdMgaPjabigdaXiabgYda8iabdMgaPjabikdaYiabgYda8iabdMgaPjabiodaZiabgYda8iabdMgaPjabisda0iabgsMiJkabdkhaYbqab0GaeyyeIuoaaaa@6AB5@

I(1,2)|3,4|=∑0≤i1<i2<i3<i4≤rWr(ui1,ui2)|Wr(ui3,ui4)|
 MathType@MTEF@5@5@+=feaafiart1ev1aaatCvAUfKttLearuWrP9MDH5MBPbIqV92AaeXatLxBI9gBaebbnrfifHhDYfgasaacH8akY=wiFfYdH8Gipec8Eeeu0xXdbba9frFj0=OqFfea0dXdd9vqai=hGuQ8kuc9pgc9s8qqaq=dirpe0xb9q8qiLsFr0=vr0=vr0dc8meaabaqaciaacaGaaeqabaqabeGadaaakeaacqWGjbqsdaWgaaWcbaGaeiikaGIaeGymaeJaeiilaWIaeGOmaiJaeiykaKIaeiiFaWNaeG4mamJaeiilaWIaeGinaqJaeiiFaWhabeaakiabg2da9maaqafabaGaem4vaCLaemOCaiNaeiikaGIaemyDau3aaSbaaSqaaiabdMgaPjabigdaXaqabaGccqGGSaalcqWG1bqDdaWgaaWcbaGaemyAaKMaeGOmaidabeaakiabcMcaPiabcYha8jabdEfaxjabdkhaYjabcIcaOiabdwha1naaBaaaleaacqWGPbqAcqaIZaWmaeqaaOGaeiilaWIaemyDau3aaSbaaSqaaiabdMgaPjabisda0aqabaGccqGGPaqkcqGG8baFaSqaaiabicdaWiabgsMiJkabdMgaPjabigdaXiabgYda8iabdMgaPjabikdaYiabgYda8iabdMgaPjabiodaZiabgYda8iabdMgaPjabisda0iabgsMiJkabdkhaYbqab0GaeyyeIuoaaaa@6AB5@

I|1,2||3,4|=∑0≤i1<i2<i3<i4≤r|Wr(ui1,ui2)||Wr(ui3,ui4)|
 MathType@MTEF@5@5@+=feaafiart1ev1aaatCvAUfKttLearuWrP9MDH5MBPbIqV92AaeXatLxBI9gBaebbnrfifHhDYfgasaacH8akY=wiFfYdH8Gipec8Eeeu0xXdbba9frFj0=OqFfea0dXdd9vqai=hGuQ8kuc9pgc9s8qqaq=dirpe0xb9q8qiLsFr0=vr0=vr0dc8meaabaqaciaacaGaaeqabaqabeGadaaakeaacqWGjbqsdaWgaaWcbaGaeiiFaWNaeGymaeJaeiilaWIaeGOmaiJaeiiFaWNaeiiFaWNaeG4mamJaeiilaWIaeGinaqJaeiiFaWhabeaakiabg2da9maaqafabaGaeiiFaWNaem4vaCLaemOCaiNaeiikaGIaemyDau3aaSbaaSqaaiabdMgaPjabigdaXaqabaGccqGGSaalcqWG1bqDdaWgaaWcbaGaemyAaKMaeGOmaidabeaakiabcMcaPiabcYha8jabcYha8jabdEfaxjabdkhaYjabcIcaOiabdwha1naaBaaaleaacqWGPbqAcqaIZaWmaeqaaOGaeiilaWIaemyDau3aaSbaaSqaaiabdMgaPjabisda0aqabaGccqGGPaqkcqGG8baFaSqaaiabicdaWiabgsMiJkabdMgaPjabigdaXiabgYda8iabdMgaPjabikdaYiabgYda8iabdMgaPjabiodaZiabgYda8iabdMgaPjabisda0iabgsMiJkabdkhaYbqab0GaeyyeIuoaaaa@6F03@

I(1,3)(2,4)=∑0≤i1<i2<i3<i4≤rWr(ui1,ui3)  Wr(ui2,ui4)
 MathType@MTEF@5@5@+=feaafiart1ev1aaatCvAUfKttLearuWrP9MDH5MBPbIqV92AaeXatLxBI9gBamXvP5wqSXMqHnxAJn0BKvguHDwzZbqegyvzYrwyUfgarqqtubsr4rNCHbGeaGqiA8vkIkVAFgIELiFeLkFeLk=iY=Hhbbf9v8qqaqFr0xc9pk0xbba9q8WqFfeaY=biLkVcLq=JHqVepeea0=as0db9vqpepesP0xe9Fve9Fve9GapdbaqaaeGacaGaaiaabeqaamqadiabaaGcbaGaemysaK0aaSbaaSqaaiabcIcaOiabigdaXiabcYcaSiabiodaZiabcMcaPiabcIcaOiabikdaYiabcYcaSiabisda0iabcMcaPaqabaGccqGH9aqpdaaeqbqaaiabdEfaxjabdkhaYjabcIcaOiabdwha1naaBaaaleaacqWGPbqAcqaIXaqmaeqaaOGaeiilaWIaemyDau3aaSbaaSqaaiabdMgaPjabiodaZaqabaGccqGGPaqkcaaMc8UaaGPaVlabdEfaxjabdkhaYjabcIcaOiabdwha1naaBaaaleaacqWGPbqAcqaIYaGmaeqaaOGaeiilaWIaemyDau3aaSbaaSqaaiabdMgaPjabisda0aqabaGccqGGPaqkaSqaaiabicdaWiabgsMiJkabdMgaPjabigdaXiabgYda8iabdMgaPjabikdaYiabgYda8iabdMgaPjabiodaZiabgYda8iabdMgaPjabisda0iabgsMiJkabdkhaYbqab0GaeyyeIuoaaaa@79BC@

I|1,3|(2,4)=∑0≤i1<i2<i3<i4≤r|Wr(ui1,ui3)|Wr(ui2,ui4)
 MathType@MTEF@5@5@+=feaafiart1ev1aaatCvAUfKttLearuWrP9MDH5MBPbIqV92AaeXatLxBI9gBaebbnrfifHhDYfgasaacH8akY=wiFfYdH8Gipec8Eeeu0xXdbba9frFj0=OqFfea0dXdd9vqai=hGuQ8kuc9pgc9s8qqaq=dirpe0xb9q8qiLsFr0=vr0=vr0dc8meaabaqaciaacaGaaeqabaqabeGadaaakeaacqWGjbqsdaWgaaWcbaGaeiiFaWNaeGymaeJaeiilaWIaeG4mamJaeiiFaWNaeiikaGIaeGOmaiJaeiilaWIaeGinaqJaeiykaKcabeaakiabg2da9maaqafabaGaeiiFaWNaem4vaCLaemOCaiNaeiikaGIaemyDau3aaSbaaSqaaiabdMgaPjabigdaXaqabaGccqGGSaalcqWG1bqDdaWgaaWcbaGaemyAaKMaeG4mamdabeaakiabcMcaPiabcYha8jabdEfaxjabdkhaYjabcIcaOiabdwha1naaBaaaleaacqWGPbqAcqaIYaGmaeqaaOGaeiilaWIaemyDau3aaSbaaSqaaiabdMgaPjabisda0aqabaGccqGGPaqkaSqaaiabicdaWiabgsMiJkabdMgaPjabigdaXiabgYda8iabdMgaPjabikdaYiabgYda8iabdMgaPjabiodaZiabgYda8iabdMgaPjabisda0iabgsMiJkabdkhaYbqab0GaeyyeIuoaaaa@6AB5@

I(1,3)|2,4|=∑0≤i1<i2<i3<i4≤rWr(ui1,ui3)|Wr(ui2,ui4)|
 MathType@MTEF@5@5@+=feaafiart1ev1aaatCvAUfKttLearuWrP9MDH5MBPbIqV92AaeXatLxBI9gBaebbnrfifHhDYfgasaacH8akY=wiFfYdH8Gipec8Eeeu0xXdbba9frFj0=OqFfea0dXdd9vqai=hGuQ8kuc9pgc9s8qqaq=dirpe0xb9q8qiLsFr0=vr0=vr0dc8meaabaqaciaacaGaaeqabaqabeGadaaakeaacqWGjbqsdaWgaaWcbaGaeiikaGIaeGymaeJaeiilaWIaeG4mamJaeiykaKIaeiiFaWNaeGOmaiJaeiilaWIaeGinaqJaeiiFaWhabeaakiabg2da9maaqafabaGaem4vaCLaemOCaiNaeiikaGIaemyDau3aaSbaaSqaaiabdMgaPjabigdaXaqabaGccqGGSaalcqWG1bqDdaWgaaWcbaGaemyAaKMaeG4mamdabeaakiabcMcaPiabcYha8jabdEfaxjabdkhaYjabcIcaOiabdwha1naaBaaaleaacqWGPbqAcqaIYaGmaeqaaOGaeiilaWIaemyDau3aaSbaaSqaaiabdMgaPjabisda0aqabaGccqGGPaqkcqGG8baFaSqaaiabicdaWiabgsMiJkabdMgaPjabigdaXiabgYda8iabdMgaPjabikdaYiabgYda8iabdMgaPjabiodaZiabgYda8iabdMgaPjabisda0iabgsMiJkabdkhaYbqab0GaeyyeIuoaaaa@6AB5@

I|1,3||2,4|=∑0≤i1<i2<i3<i4≤r|Wr(ui1,ui3)||Wr(ui2,ui4)|
 MathType@MTEF@5@5@+=feaafiart1ev1aaatCvAUfKttLearuWrP9MDH5MBPbIqV92AaeXatLxBI9gBaebbnrfifHhDYfgasaacH8akY=wiFfYdH8Gipec8Eeeu0xXdbba9frFj0=OqFfea0dXdd9vqai=hGuQ8kuc9pgc9s8qqaq=dirpe0xb9q8qiLsFr0=vr0=vr0dc8meaabaqaciaacaGaaeqabaqabeGadaaakeaacqWGjbqsdaWgaaWcbaGaeiiFaWNaeGymaeJaeiilaWIaeG4mamJaeiiFaWNaeiiFaWNaeGOmaiJaeiilaWIaeGinaqJaeiiFaWhabeaakiabg2da9maaqafabaGaeiiFaWNaem4vaCLaemOCaiNaeiikaGIaemyDau3aaSbaaSqaaiabdMgaPjabigdaXaqabaGccqGGSaalcqWG1bqDdaWgaaWcbaGaemyAaKMaeG4mamdabeaakiabcMcaPiabcYha8jabcYha8jabdEfaxjabdkhaYjabcIcaOiabdwha1naaBaaaleaacqWGPbqAcqaIYaGmaeqaaOGaeiilaWIaemyDau3aaSbaaSqaaiabdMgaPjabisda0aqabaGccqGGPaqkcqGG8baFaSqaaiabicdaWiabgsMiJkabdMgaPjabigdaXiabgYda8iabdMgaPjabikdaYiabgYda8iabdMgaPjabiodaZiabgYda8iabdMgaPjabisda0iabgsMiJkabdkhaYbqab0GaeyyeIuoaaaa@6F03@

I(1,4)(2,3)=∑0≤i1<i2<i3<i4≤rWr(ui1,ui4)Wr(ui2,ui3)
 MathType@MTEF@5@5@+=feaafiart1ev1aaatCvAUfKttLearuWrP9MDH5MBPbIqV92AaeXatLxBI9gBaebbnrfifHhDYfgasaacH8akY=wiFfYdH8Gipec8Eeeu0xXdbba9frFj0=OqFfea0dXdd9vqai=hGuQ8kuc9pgc9s8qqaq=dirpe0xb9q8qiLsFr0=vr0=vr0dc8meaabaqaciaacaGaaeqabaqabeGadaaakeaacqWGjbqsdaWgaaWcbaGaeiikaGIaeGymaeJaeiilaWIaeGinaqJaeiykaKIaeiikaGIaeGOmaiJaeiilaWIaeG4mamJaeiykaKcabeaakiabg2da9maaqafabaGaem4vaCLaemOCaiNaeiikaGIaemyDau3aaSbaaSqaaiabdMgaPjabigdaXaqabaGccqGGSaalcqWG1bqDdaWgaaWcbaGaemyAaKMaeGinaqdabeaakiabcMcaPiabdEfaxjabdkhaYjabcIcaOiabdwha1naaBaaaleaacqWGPbqAcqaIYaGmaeqaaOGaeiilaWIaemyDau3aaSbaaSqaaiabdMgaPjabiodaZaqabaGccqGGPaqkaSqaaiabicdaWiabgsMiJkabdMgaPjabigdaXiabgYda8iabdMgaPjabikdaYiabgYda8iabdMgaPjabiodaZiabgYda8iabdMgaPjabisda0iabgsMiJkabdkhaYbqab0GaeyyeIuoaaaa@6667@

I|1,4|(2,3)=∑0≤i1<i2<i3<i4≤r|Wr(ui1,ui4)|Wr(ui2,ui3)
 MathType@MTEF@5@5@+=feaafiart1ev1aaatCvAUfKttLearuWrP9MDH5MBPbIqV92AaeXatLxBI9gBaebbnrfifHhDYfgasaacH8akY=wiFfYdH8Gipec8Eeeu0xXdbba9frFj0=OqFfea0dXdd9vqai=hGuQ8kuc9pgc9s8qqaq=dirpe0xb9q8qiLsFr0=vr0=vr0dc8meaabaqaciaacaGaaeqabaqabeGadaaakeaacqWGjbqsdaWgaaWcbaGaeiiFaWNaeGymaeJaeiilaWIaeGinaqJaeiiFaWNaeiikaGIaeGOmaiJaeiilaWIaeG4mamJaeiykaKcabeaakiabg2da9maaqafabaGaeiiFaWNaem4vaCLaemOCaiNaeiikaGIaemyDau3aaSbaaSqaaiabdMgaPjabigdaXaqabaGccqGGSaalcqWG1bqDdaWgaaWcbaGaemyAaKMaeGinaqdabeaakiabcMcaPiabcYha8jabdEfaxjabdkhaYjabcIcaOiabdwha1naaBaaaleaacqWGPbqAcqaIYaGmaeqaaOGaeiilaWIaemyDau3aaSbaaSqaaiabdMgaPjabiodaZaqabaGccqGGPaqkaSqaaiabicdaWiabgsMiJkabdMgaPjabigdaXiabgYda8iabdMgaPjabikdaYiabgYda8iabdMgaPjabiodaZiabgYda8iabdMgaPjabisda0iabgsMiJkabdkhaYbqab0GaeyyeIuoaaaa@6AB5@

I(1,4)|2,3|=∑0≤i1<i2<i3<i4≤rWr(ui1,ui4)|Wr(ui2,ui3)|
 MathType@MTEF@5@5@+=feaafiart1ev1aaatCvAUfKttLearuWrP9MDH5MBPbIqV92AaeXatLxBI9gBaebbnrfifHhDYfgasaacH8akY=wiFfYdH8Gipec8Eeeu0xXdbba9frFj0=OqFfea0dXdd9vqai=hGuQ8kuc9pgc9s8qqaq=dirpe0xb9q8qiLsFr0=vr0=vr0dc8meaabaqaciaacaGaaeqabaqabeGadaaakeaacqWGjbqsdaWgaaWcbaGaeiikaGIaeGymaeJaeiilaWIaeGinaqJaeiykaKIaeiiFaWNaeGOmaiJaeiilaWIaeG4mamJaeiiFaWhabeaakiabg2da9maaqafabaGaem4vaCLaemOCaiNaeiikaGIaemyDau3aaSbaaSqaaiabdMgaPjabigdaXaqabaGccqGGSaalcqWG1bqDdaWgaaWcbaGaemyAaKMaeGinaqdabeaakiabcMcaPiabcYha8jabdEfaxjabdkhaYjabcIcaOiabdwha1naaBaaaleaacqWGPbqAcqaIYaGmaeqaaOGaeiilaWIaemyDau3aaSbaaSqaaiabdMgaPjabiodaZaqabaGccqGGPaqkcqGG8baFaSqaaiabicdaWiabgsMiJkabdMgaPjabigdaXiabgYda8iabdMgaPjabikdaYiabgYda8iabdMgaPjabiodaZiabgYda8iabdMgaPjabisda0iabgsMiJkabdkhaYbqab0GaeyyeIuoaaaa@6AB5@

I|1,4||2,3|=∑0≤i1<i2<i3<i4≤r|Wr(ui1,ui4)||Wr(ui2,ui3)|
 MathType@MTEF@5@5@+=feaafiart1ev1aaatCvAUfKttLearuWrP9MDH5MBPbIqV92AaeXatLxBI9gBaebbnrfifHhDYfgasaacH8akY=wiFfYdH8Gipec8Eeeu0xXdbba9frFj0=OqFfea0dXdd9vqai=hGuQ8kuc9pgc9s8qqaq=dirpe0xb9q8qiLsFr0=vr0=vr0dc8meaabaqaciaacaGaaeqabaqabeGadaaakeaacqWGjbqsdaWgaaWcbaGaeiiFaWNaeGymaeJaeiilaWIaeGinaqJaeiiFaWNaeiiFaWNaeGOmaiJaeiilaWIaeG4mamJaeiiFaWhabeaakiabg2da9maaqafabaGaeiiFaWNaem4vaCLaemOCaiNaeiikaGIaemyDau3aaSbaaSqaaiabdMgaPjabigdaXaqabaGccqGGSaalcqWG1bqDdaWgaaWcbaGaemyAaKMaeGinaqdabeaakiabcMcaPiabcYha8jabcYha8jabdEfaxjabdkhaYjabcIcaOiabdwha1naaBaaaleaacqWGPbqAcqaIYaGmaeqaaOGaeiilaWIaemyDau3aaSbaaSqaaiabdMgaPjabiodaZaqabaGccqGGPaqkcqGG8baFaSqaaiabicdaWiabgsMiJkabdMgaPjabigdaXiabgYda8iabdMgaPjabikdaYiabgYda8iabdMgaPjabiodaZiabgYda8iabdMgaPjabisda0iabgsMiJkabdkhaYbqab0GaeyyeIuoaaaa@6F03@

#### Selecting the models

We use a procedure very similar to that of the SSEF method to obtain a representative set of fragments. The SSEF method derives its representative set of structural fragments from the SCOP classification database [20]. Similarly to CATH, SCOP is an hierarchical classification database that organizes protein domains into four classification levels: Class, Fold, Superfamily and Family. For the SEGF method, we first extract all backbone segments from protein domains in the SCOP fold dataset, a set of structures that represent every fold in the SCOP database version 1.65 [20]. The extracted segments are clustered with a *k*-means clustering algorithm to obtain a total of *p *(in our case *p *= 300) clusters. From each cluster we select one backbone segment, the one closest to the cluster center, for our set of models. For the purpose of clustering, each backbone segment is represented by a point in *R*^14^. Before the clustering is carried out we normalize the data points by applying a standard normalization procedure to each of the fourteen dimensions, where values in dimension *i *are normalized by subtracting their mean and dividing by their standard deviation.

#### Footprint computation

The footprint computation step is also very similar to that of the SSEF method. The structural footprint ***f*_*Q *_**of a structure *Q *is computed by having each backbone segment in *Q *distribute a count of one among a subset of models, where the most similar models get the biggest share. This procedure is formalized below:

fQ=(f1Q,...,fpQ)
 MathType@MTEF@5@5@+=feaafiart1ev1aaatCvAUfKttLearuWrP9MDH5MBPbIqV92AaeXatLxBI9gBaebbnrfifHhDYfgasaacH8akY=wiFfYdH8Gipec8Eeeu0xXdbba9frFj0=OqFfea0dXdd9vqai=hGuQ8kuc9pgc9s8qqaq=dirpe0xb9q8qiLsFr0=vr0=vr0dc8meaabaqaciaacaGaaeqabaqabeGadaaakeaaieWacqWFMbGzdaWgaaWcbaGae8xuaefabeaakiabg2da9iabcIcaOiabdAgaMnaaDaaaleaacqaIXaqmaeaacqWGrbquaaGccqGGSaalcqGGUaGlcqGGUaGlcqGGUaGlcqGGSaalcqWGMbGzdaqhaaWcbaGaemiCaahabaGaemyuaefaaOGaeiykaKcaaa@3E51@

fiQ=∑s:d(s,mi)<γc(s,mi)
 MathType@MTEF@5@5@+=feaafiart1ev1aaatCvAUfKttLearuWrP9MDH5MBPbIqV92AaeXatLxBI9gBaebbnrfifHhDYfgasaacH8akY=wiFfYdH8Gipec8Eeeu0xXdbba9frFj0=OqFfea0dXdd9vqai=hGuQ8kuc9pgc9s8qqaq=dirpe0xb9q8qiLsFr0=vr0=vr0dc8meaabaqaciaacaGaaeqabaqabeGadaaakeaacqWGMbGzdaqhaaWcbaGaemyAaKgabaGaemyuaefaaOGaeyypa0ZaaabuaeaacqWGJbWycqGGOaakcqWGZbWCcqGGSaalcqWGTbqBdaWgaaWcbaGaemyAaKgabeaakiabcMcaPaWcbaGaem4CamNaeiOoaOJaemizaqMaeiikaGIaem4CamNaeiilaWIaemyBa02aaSbaaWqaaiabdMgaPbqabaWccqGGPaqkcqGH8aapiiGacqWFZoWzaeqaniabggHiLdaaaa@4990@

c(s,mi)=exp⁡(−d(s,mi)2/a)∑mjexp⁡(−d(s,mj)2/a)
 MathType@MTEF@5@5@+=feaafiart1ev1aaatCvAUfKttLearuWrP9MDH5MBPbIqV92AaeXatLxBI9gBaebbnrfifHhDYfgasaacH8akY=wiFfYdH8Gipec8Eeeu0xXdbba9frFj0=OqFfea0dXdd9vqai=hGuQ8kuc9pgc9s8qqaq=dirpe0xb9q8qiLsFr0=vr0=vr0dc8meaabaqaciaacaGaaeqabaqabeGadaaakeaacqWGJbWycqGGOaakcqWGZbWCcqGGSaalcqWGTbqBdaWgaaWcbaGaemyAaKgabeaakiabcMcaPiabg2da9maalaaabaGagiyzauMaeiiEaGNaeiiCaaNaeiikaGIaeyOeI0IaemizaqMaeiikaGIaem4CamNaeiilaWIaemyBa02aaSbaaSqaaiabdMgaPbqabaGccqGGPaqkdaahaaWcbeqaaiabikdaYaaakiabc+caViabdggaHjabcMcaPaqaamaaqababaGagiyzauMaeiiEaGNaeiiCaaNaeiikaGIaeyOeI0IaemizaqMaeiikaGIaem4CamNaeiilaWIaemyBa02aaSbaaSqaaiabdQgaQbqabaGccqGGPaqkdaahaaWcbeqaaiabikdaYaaakiabc+caViabdggaHjabcMcaPaWcbaGaemyBa02aaSbaaWqaaiabdQgaQbqabaaaleqaniabggHiLdaaaaaa@5FC9@

*s *is a structural fragment of *Q*

*c*(*s*, *m*_*i*_) is a contribution of *s *to model *m*_*i*_

*d*(*s*, *m*_*i*_) is the distance between *s *and a model *m*_*i *_(Euclidean distance between the corresponding points in *R*^14^)

*a *is a scale factor

*γ *is a threshold

A structural fragment *s *contributes to a model *m *only if they are similar enough, i.e., the distance *d*(*s*, *m*) is below a certain threshold *γ*. The value of this threshold and the scale factor *a *are determined from the distribution of distances of a structural fragment to the closest model [see Additional file 3].

#### Computing structural similarity

Once footprints are computed, a distance between two protein domains is measured by the Pearson correlation coefficient of their footprints ***f*_*Q *_**and ***f*_*P*_**:

∑i=1p(fiQ−μQ)(fiP−μP)∑i=1p(fiQ−μQ)2∑i=1p(fiP−μP)2
 MathType@MTEF@5@5@+=feaafiart1ev1aaatCvAUfKttLearuWrP9MDH5MBPbIqV92AaeXatLxBI9gBaebbnrfifHhDYfgasaacH8akY=wiFfYdH8Gipec8Eeeu0xXdbba9frFj0=OqFfea0dXdd9vqai=hGuQ8kuc9pgc9s8qqaq=dirpe0xb9q8qiLsFr0=vr0=vr0dc8meaabaqaciaacaGaaeqabaqabeGadaaakeaadaWcaaqaamaaqadabaGaeiikaGIaemOzay2aa0baaSqaaiabdMgaPbqaaiabdgfarbaakiabgkHiTGGaciab=X7aTnaaBaaaleaacqWGrbquaeqaaOGaeiykaKIaeiikaGIaemOzay2aa0baaSqaaiabdMgaPbqaaiabdcfaqbaakiabgkHiTiab=X7aTnaaBaaaleaacqWGqbauaeqaaOGaeiykaKcaleaacqWGPbqAcqGH9aqpcqaIXaqmaeaacqWGWbaCa0GaeyyeIuoaaOqaamaakaaabaWaaabmaeaacqGGOaakcqWGMbGzdaqhaaWcbaGaemyAaKgabaGaemyuaefaaOGaeyOeI0Iae8hVd02aaSbaaSqaaiabdgfarbqabaGccqGGPaqkdaahaaWcbeqaaiabikdaYaaaaeaacqWGPbqAcqGH9aqpcqaIXaqmaeaacqWGWbaCa0GaeyyeIuoaaSqabaGcdaGcaaqaamaaqadabaGaeiikaGIaemOzay2aa0baaSqaaiabdMgaPbqaaiabdcfaqbaakiabgkHiTiab=X7aTnaaBaaaleaacqWGqbauaeqaaOGaeiykaKYaaWbaaSqabeaacqaIYaGmaaaabaGaemyAaKMaeyypa0JaeGymaedabaGaemiCaahaniabggHiLdaaleqaaaaaaaa@6A72@

where *μ*_*Q *_and *μ*_*P *_are the means of ***f*_*Q *_**and ***f*_*P*_**, respectively.

### Learning the linear combination coefficients with an SVM

A new structural similarity score between the query and a database domain can be defined as a linear combination of original similarity scores:

*sim*_COMB _= *w*_SSEF _*sim*_SSEF _+ *w*_SEGF _*sim*_SEGF _+ *w*_LFF _*sim*_LFF _- *w*_0_.

The coefficients (*w*_SSEF_, *w*_SEGF_, and *w*_LFF_) are learned with an SVM from a set of positive and negative examples. For each well-populated superfamily we selected uniformly at random 10 pairs of domains where both domains are from the superfamily in the set of positive examples, and 10 pairs of domains where one domain is from the superfamily and another domain is from a different fold in the set of negative examples. Therefore each set contains 1,330 domain pairs, 10 pairs for each of the 133 well-populated superfamilies [see Additional file 4]. We used the SVMLight implementation [21] of the SVM learning algorithm with default parameters. The set of coefficients learned is summarized in Table 3.

**Table 3:** Coefficient values used in linear combination strategy. Coefficient values learned with SVM for the four combinations: SSEF+SEGF+LFF, SSEF+SEGF, SSEF+LFF, and SEGF+LFF.

	*w*_SSEF_	*w*_SEGF_	*w*_LFF_	*w*_0_
SSEF+SEGF+LFF	6.08	3.16	2.85	8.57
SSEF+SEGF	7.28	3.72	N/A	7.26
SSEF+LFF	7.48	N/A	4.18	8.56
SEGF+LFF	N/A	6.34	6.38	9.76

### Computing agreement values for superfamilies

Given a classification group, the method's agreement with the CATH hierarchy for this group is equal to an average agreement value taken over all well-populated superfamilies that are members of the group. For a member superfamily the agreement value is measured by a ROC score taken over the list of well-populated superfamilies ranked by their affinity to the superfamily, where the positives are superfamilies in the same classification group and the negatives are superfamilies outside the group. To separate classification groups at different levels of the hierarchy, we further restrict the set of positives in our ROC score computation: at the Class level the positives are superfamilies within the same class but different architecture groups than the query superfamily, at the Architecture level the positives are within the same architecture but different fold groups, and at the Fold level the positives are within the same fold group. Since not every architecture group contains at least two superfamilies from different fold groups and not every fold group contains at least two superfamilies, the agreement values are not available for all the architectures and folds spanned by the set of well-populated superfamilies.

### Programs

For the LFF method, we obtained the set of models from the authors of the LFF method. We computed footprints and distances as described in [5]. We implemented the prototype of the SEGF method in Python using the BioPython suite of packages [22]. The Python code and auxiliary files necessary to compute: (i) the SEGF footprint from a PDB file of a structure; (ii) the SSEF footprint from a PDB file of a structure; (iii) structural similarity score given PDB files of structures using either SSEF, SEGF or SSEF+SEGF methods, are given as supplementary material [see Additional file 3].

## Authors' contributions

EZ provided input on the design of the SEGF method, combination strategies, affinity scores, and thresholded affinity graph, participated in data analysis, carried out the experiments, and drafted the manuscript. RI and WJW provided input on the application of machine learning techniques to combine the methods' output. DPO provided input on the design of combination strategies and thresholded affinity graph, participated in data analysis, and helped to draft the manuscript. TMP proposed the study, provided input on the design of the SEGF method, combination strategies, and affinity scores, participated in the data analysis, and helped to draft the manuscript. All authors read and approved the final manuscript.

## Supplementary Material

Additional file 3 The Python code for the SSEF and SEGF methods. An archive that contains the Python code and auxiliary files necessary to compute: (i) the SEGF footprint from a PDB file of a structure; (ii) the SSEF footprint from a PDB file of a structure; (iii) structural similarity score given PDB files of structures using either SSEF, SEGF or SSEF+SEGF method. Click here for file

Additional file 4 The set of training examples used to learn linear combination coefficients. A text file that contains the set of training examples used to learn linear combination coefficients. Click here for file

Additional file 1 ROC scores for all superfamilies included in the study. An Excel file with performance, in terms of *ROC*_300 _scores, for all superfamilies included in the study and across different methods: SSEF, SEGF, LFF, voting, SSEF+SEGF, SSEF+LFF, SEGF+LFF, SSEF+SEGF+LFF. Click here for file

Additional file 2 Affinity graphs and affinity scores for all three methods. An archive that contains the affinity graphs for all three methods. The graphs are given in Cytoscape's GML format. We also include a separate file for each method giving the affinity scores. Click here for file
