# Supplementary figures and images for: Structural footprinting in protein structure comparison: the impact of structural fragments
Source: BMC Struct Biol. 2007 Aug 9;7:53. doi: 10.1186/1472-6807-7-53 (PMC2082327; doi:10.1186/1472-6807-7-53)

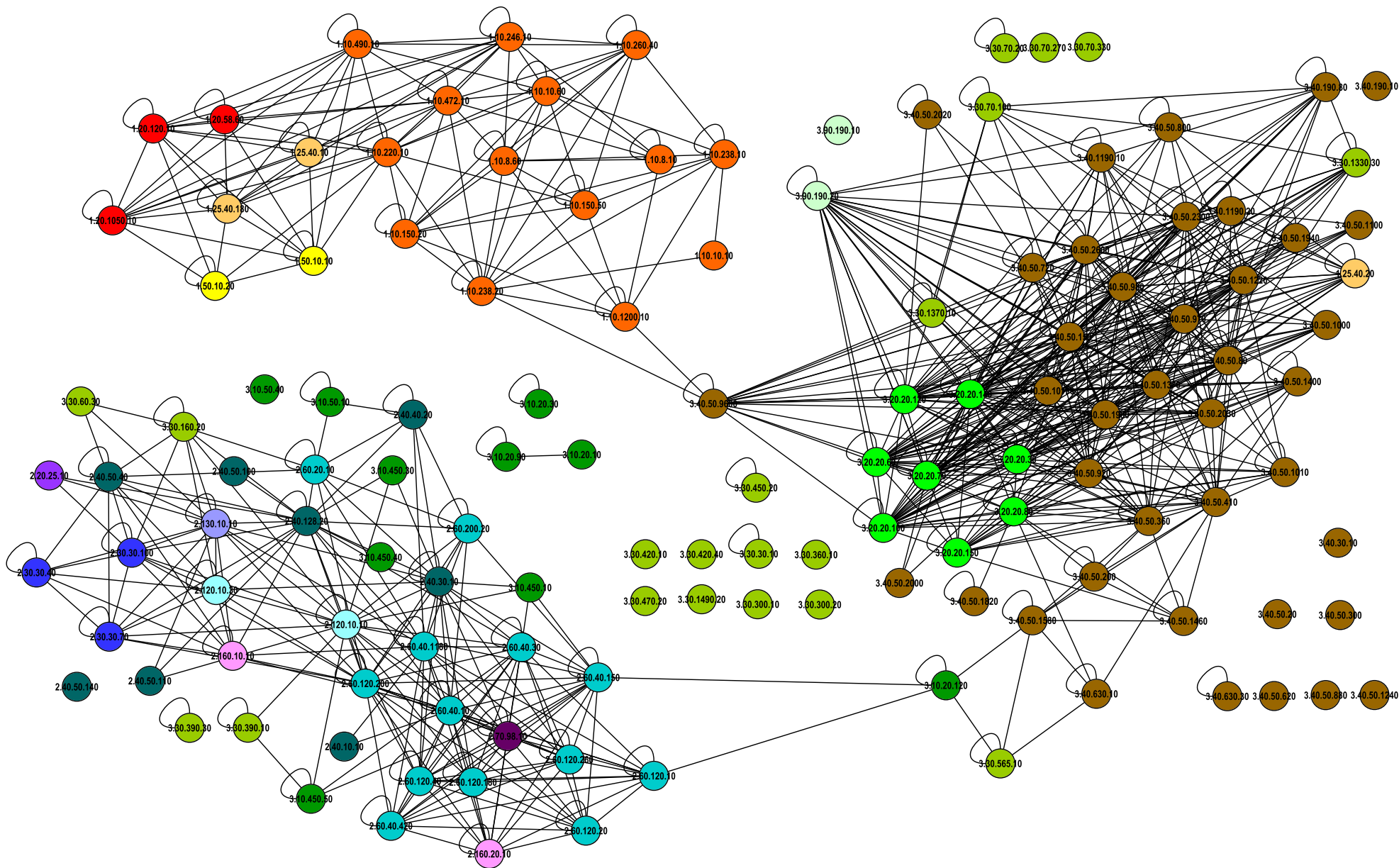

Supplement: Additional file 2 — Affinity graphs and affinity scores for all three methods. An archive that contains the affinity graphs for all three methods. The graphs are given in Cytoscape's GML format. We also include a separate file for each method giving the affinity scores. [file 1472-6807-7-53-S2.zip › affinity_graph_seg.pdf]
